# GS-5734 and its parent nucleoside analog inhibit Filo-, Pneumo-, and Paramyxoviruses

**DOI:** 10.1038/srep43395

**Published:** 2017-03-06

**Authors:** Michael K. Lo, Robert Jordan, Aaron Arvey, Jawahar Sudhamsu, Punya Shrivastava-Ranjan, Anne L. Hotard, Mike Flint, Laura K. McMullan, Dustin Siegel, Michael O. Clarke, Richard L. Mackman, Hon C. Hui, Michel Perron, Adrian S. Ray, Tomas Cihlar, Stuart T. Nichol, Christina F. Spiropoulou

**Affiliations:** 1 Centers for Disease Control and Prevention, Atlanta, Georgia, USA; 2 Gilead Sciences, Inc. Foster City, California, USA

## Abstract

GS-5734 is a monophosphate prodrug of an adenosine nucleoside analog that showed therapeutic efficacy in a non-human primate model of Ebola virus infection. It has been administered under compassionate use to two Ebola patients, both of whom survived, and is currently in Phase 2 clinical development for treatment of Ebola virus disease. Here we report the antiviral activities of GS-5734 and the parent nucleoside analog across multiple virus families, providing evidence to support new indications for this compound against human viruses of significant public health concern.

Viruses belonging to the families *Paramyxoviridae, Coronaviridae* and *Filoviridae* include zoonotic and human pathogens that are of significant public health concern, ranging from vaccine-preventable diseases such as Measles (MV) and Mumps (MuV) viruses to highly pathogenic viruses such as Nipah (NiV), Middle East Respiratory Syndrome (MERS) and Ebola (EBOV) viruses, for which there are currently no approved therapeutics for human use. A broad spectrum therapeutic that can target multiple virus families would have a significant impact on disease burden in endemic areas. Nucleoside analogs are a class of small-molecule antivirals which can directly inhibit viral transcription and replication by targeting the viral RNA-dependent RNA polymerase, and have been used as backbone components of combination therapies against both Human immunodeficiency virus (HIV) and Hepatitis C virus (HCV)^1^,^2^. A 1′-cyano substituted adenine nucleoside analog GS-441524 (“Nuc”) was shown to have activity against HCV, dengue virus (DenV), parainfluenza type 3 virus (hPIV3), and severe acute respiratory syndrome coronavirus (SARS-CoV)^3^. GS-5734 is a monophosphate prodrug of Nuc that has shown activity against filoviruses and coronaviruses in cell-based antiviral assays^4^. More importantly, this compound protected non-human primates from lethal Ebola virus infection when given therapeutically beginning 3 days post-inoculation, and is currently in phase 2 clinical development for treatment of Ebola virus disease.

To determine the full spectrum of activity of Nuc and GS-5734 against a wide panel of human viral pathogens, the antiviral activity was determined using a variety of cell-based assays. Nuc was active against recombinant reporter-expressing NiV^5^, EBOV^6^,^7^, and Marburg viruses (MARV)^8^, with observed 50% effective inhibition concentration (EC_50_) values between 1–3 μM (Table 1; Supplementary Figure 1, Supplementary Table 1). We confirmed the activity of Nuc against wild-type EBOV (Makona strain), NiV (Malaysian and Bangladesh genotypes) and Hendra virus (HeV) using assays which measured inhibition of virus antigen expression, virus-induced cytopathic effect (CPE) (for NiV and HeV), and virus titer (Supplementary Figures 2–4, Supplementary Table 1). The EC_50_ values derived from the different assays using wild-type viruses closely paralleled the values observed from the reporter viruses (Supplementary Table 1) (For details regarding the different assays used, please see Methods section). Moreover, at concentrations between 5–16 μM, Nuc was able to reduce infectious virus production of NiV and EBOV by greater than 5 logs (Supplementary Figure 4). To explore the range of Nuc antiviral activity across the *Paramyxoviridae* and *Pneumoviridae*^9^ families, we tested Nuc against reporter viruses from representative genera including MV^10^, hPIV3^11^, respiratory syncytial virus (RSV)^12^, human metapneumovirus^13^ (hMPV), as well as wild-type MV and MuV^14^ (Supplementary Figures 1 and 2). While EC_50_ values for Nuc against MV, hPIV3, hMPV, and RSV ranged between 0.5 to 2 μM, the EC_50_ value against MuV was consistently in the 7–12 μM range. However, when we tested Nuc against viruses from unrelated families such as *Arenaviridae* (Lassa virus (LASV)), *Rhabdoviridae* (Vesicular Stomatitis virus (VSV)), *Bunyaviridae* (Rift Valley Fever virus (RVFV)^15^, Crimean Congo Hemorrhagic Fever virus (CCHFV)^16^), and several tick-borne members of *Flaviviridae* (Alkhurma Hemorrhagic Fever virus (AHFV), Kyasanur Forest Disease virus (KFDV), Omsk Hemorrhagic Fever virus (OHFV), Tick-borne encephalitis (TBEV)), we observed little to no antiviral activity (Table 1; Supplementary Figures 1–3; Supplementary Table 1).

GS-5734 is a prodrug that is designed to deliver the nucleoside monophosphate into the cell, thereby circumventing the rate-limiting first phosphorylation step and allowing for efficient formation of the active triphosphate species. Consistent with this notion, GS-5734 exhibited 10 to 100-fold higher antiviral potency relative to Nuc when tested against reporter and wild-type filo-, pneumo-, and paramyxoviruses, yielding EC_50_ values ranging from 0.003 to 0.79 μM (Table 1, Supplementary Table 2; Supplementary Figures 6–8). Moreover, GS-5734 was able to reduce infectious virus production of NiV and EBOV by greater than 4 logs but at much lower concentrations than Nuc, reflecting the more efficient metabolism of the prodrug to the active form (Table 1, Supplementary Figure 10, Supplementary Table 2). To confirm that GS-5734 specifically inhibits viral transcription and replication, we measured GS-5734 activity against EBOV and NiV minigenome replication, and observed dose-dependent inhibition with EC_50_ values in the sub-micromolar range (Supplementary Figure 9; Supplementary Table 2). Of particular note is that compared to immortalized cells, GS-5734 had similar or even enhanced antiviral potency in primary microvascular endothelial cells and macrophages, which are major cellular targets in human infection for NiV and EBOV respectively (Supplementary Figure 10; Supplementary Table 2)^4^. As was done for Nuc, we evaluated the activity of GS-5734 across other virus families and observed significantly lower activity, with micromolar EC_50_ values against tick-borne flaviviruses and LASV, and minimal to no antiviral activity against bunyaviruses (Table 1; Supplementary Table 2; Supplementary Figures 6–8). The EC_50_ values for Nuc and GS-5734 against filo-, pneumo-, and paramyxoviruses (with the exception of MuV) were at least 16-fold and 150-fold lower than the 50% cytotoxic concentrations in the cell types tested, respectively (Table 1; Supplementary Figures 5 and 11; Supplementary Tables 1–3). While the observed antiviral activity across the families *Paramyxo*-, *Pneumo*-, *Corona*-17, and *Filoviridae* was relatively consistent (with the exception of MuV), there were variable levels of activity across the family *Flaviviridae*, with moderate activity against HCV, DenV, and Yellow Fever virus (YFV), but significantly lower to negligible activity against West Nile virus (WNV)^3^ and the tick-borne flaviviruses tested in this study.

To determine whether structural and sequence similarities of viral RNA-dependent RNA polymerases (RdRPs) would provide additional insight into the observed antiviral spectrum, we took a hybrid approach that used both structural homology and primary protein sequence information to align the secondary structure of nucleotide binding domains (NBDs) from known crystal structures of various viral RdRps. Distinct ssRNA virus families do not necessarily share a common ancestor in the traditional phylogenetic sense, thus the primary sequence and structure of RNA-dependent RNA polymerases (RdRPs) can be quite divergent. We therefore examined all available viral RdRP crystal structures and utilized secondary structure to align the nucleotide binding domains in iterative fashion by removing non-homologous regions. This resulted in a compelling structural alignment for the nucleotide binding domains, despite the overall structures of RdRPs being very different (Fig. 1B). For example, structural alignments of HCV against DenV (Fig. 1C) and VSV (Fig. 1D) RdRP nucleotide binding domains both show good overlap in the nucleotide binding regions, but have significant differences globally. This structural alignment informed the extraction of two 10-residue regions within motifs A and B that interact directly with the nucleotide^18^,^19^,^20^ (Fig. 1A). Sequence similarity clustering analysis of these regions correlated with the observed spectrum of antiviral activity across virus families (Fig. 2). For instance we observe that while HCV and DenV are both inhibited by GS-5734 and have high sequence similarity among motifs A and B, VSV which is not inhibited by GS-5734 has low sequence similarity among motifs A and B when compared to HCV and other negative strand RNA viruses susceptible to GS-5734 inhibition. Taken together, our results expand the spectrum of viruses susceptible to Nuc and GS-5734, and further define their respective antiviral activities across 7 virus families. The unique potent broad-spectrum activity of GS-5734 against paramyxoviruses, coronaviruses, and filoviruses provides an opportunity to explore the compound for the treatment of highly pathogenic infections such as NiV and MERS-CoV in non-human primates, in addition to EBOV and other filoviruses. Depending on further careful assessment of its safety and efficacy profile, GS-5734 could also be considered for the treatment of prevalent respiratory infections (RSV, hPIV3, hMPV), as well as for controlling recurring outbreaks of MV globally among under-vaccinated populations.

## Methods

### Small molecules

GS-5734 and Nuc were synthesized at Gilead Sciences, Inc., and chemical identity and sample purity were established using NMR, HRMS, and HPLC analysis^4^. To determine concentration response curves against each virus, we used either a 7-point or a 10-point 3-fold dilution series, with each compound concentration tested in quadruplicate.

### Cells

HeLa (ATCC CCL-2) and Huh7 (Apath, LLC) cells were cultivated in Dulbecco’s Minimal Essential Medium (DMEM) supplemented with 7.5% fetal bovine serum (FBS), non-essential amino acids, and penicillin/streptomycin. HEK293T/17 (ATCC CRL-11286), Vero (ATCC CCL-81), NCI-H358 (ATCC CRL-5807), and A549 (ATCC CCL-185) cells were cultivated in DMEM supplemented with 10% FBS. Primary human lung microvascular endothelial cells (HMVEC-L) cells (Lonza CC-2527) were cultivated using EGM-2 MV BulletKit (Lonza CC-3202). Human peripheral blood macrophages (Stemcell Technologies) were cultivated in Macrophage-SFM (Gibco). All cells were incubated at 37 °C in 5% CO2.

### Viruses

Nipah virus (NiV-M, Malaysian genotype; NiV-B, Bangladesh genotype; NiV-Luc2AM recombinant Malaysia genotype expressing Renilla luciferase; NiV-GFP2AM, recombinant Malaysian genotype expressing ZsGreen fluorescent protein)^5^, Hendra virus (HeV), Measles virus (MV-EZ, Edmonston-Zagreb vaccine strain; rMVEZEGFP(3), recombinant Measles Edmonston-Zagreb strain expressing enhanced green fluorescent protein)^10^, Mumps virus (MuV, Iowa 2006), Ebola virus (EBOV, Makona variant; EBOV-GFP, recombinant Mayinga variant expressing green-fluorescent protein; EBOV-GLuc, recombinant Mayinga variant expressing Gaussia luciferase)^6^,^21^; Marburg virus (MARV-GFP, recombinant Bat 371 variant expressing enhanced green fluorescent protein; MARV-Gluc, recombinant Bat 371 variant expressing Gaussia luciferase)^8^,^21^, Lassa virus (LASV, Josiah), Andes virus (ANDV, Chile 9717869), Rift Valley Fever Virus (RVFV-GFP, ZH501)^22^, Alkhurma Hemorrhagic Fever virus (AHFV, 20030001), Omsk Hemorrhagic Fever virus (OHFV, Bogoluvovska), Kyasanur Forest Disease virus (KFDV, P9605), Tick-borne Encephalitis virus (TBEV, Hypr), and Vesicular Stomatitis virus (VSV, New Jersey) were propagated in either Vero E6 (ATCC CRL-1586) or Vero (ATCC CCL-81) cells, and were quantitated by 50% tissue culture infections dose (TCID_50_) assay using the Reed and Muench method^23^. EBOV-ZsG (recombinant Makona variant)^7^ was propagated and quantitated as above using Huh7 cells. The rgRSV was propagated and quantitated as above using HeLa cells^12^. Both hMPV-GFP (CAN97-83)^13^ and hPIV3-GFP (JS)^11^ were obtained from ViraTree.

### Reporter virus assays

All viruses expressing either fluorescent (eGFP, ZsGreen) or bioluminescent (Renilla luciferase, Gaussia luciferase) proteins were assayed for fluorescence/luminescence using an HD1 Synergy plate reader (Biotek). 50% effective concentrations (EC_50_) were calculated using four-parameter variable slope non-linear regression fitting of mean values of assays performed in quadruplicate. NiV-GFP2AM and NiV-Luc2AM assays were performed in HeLa or 293 T/17 cells. hPIV3-GFP, rgRSV224, rMV^EZ^(3)GFP assays were performed in HeLa cells. hMPV-GFP assay was performed in Vero cells. EBOV-GFP, EBOV-GLuc, EBOV-ZsG, MARV-GFP, MARV-GLuc, and RVFV-GFP assays were performed in Huh7 cells. Cells were seeded at 10,000 cells per well in black opaque 96-well plates, and compounds were added to the assay plates. Assay plates were transferred to the BSL-4 suite (where appropriate), and infected with 0.25 TCID_50_ per cell of the respective virus, and were read between 48 and 144 hours post-infection (hpi) for fluorescence or luminescence, using the respectively appropriate reagents (Renilla-Glo reagent (Promega); Biolux Gaussia luciferase stabilized reagent (New England Biolabs)) depending on the virus used.

### Viral antigen reduction assays

Levels of viral replication was measured by either a fluorescence or chemiluminescence-based immuno-staining method using antibodies against the corresponding viral antigen(s)^24^,^25^. Fluorescence or luminescence was measured using an HD1 Synergy plate reader (Biotek). Untreated infected cell control values (after subtraction of reference values) were set at 100% viral antigen. EC_50_ values were calculated using four-parameter variable slope non-linear regression fitting of mean values from assays performed in quadruplicate. NiV-M, NiV-B, HeV, MV-EZ, and MuV assays were performed in HeLa cells. EBOV, ANDV, LASV, and CCHFV assays were performed in Huh7 cells. Cells were seeded at 10,000 cells per well in either black opaque, clear-bottom (for fluorescence-based assay) or white (for chemiluminescence-based assay) opaque 96-well plates, and compounds were added to the assay plates. Assay plates were transferred to the BSL-4 suite (where appropriate) and infected with 0.25 TCID_50_ per cell with respective virus, and were fixed at 24–72 hpi with 10% formalin supplemented with 0.2% Triton-X 100 detergent before downstream staining with primary antibody specific for the respective virus, and secondary antibodies (conjugated with Dylight-488 or horseradish peroxidase). Primary antibodies used for each respective virus are indicated in the following: NiV-M, NiV-B, and HeV: [1A11C1]^26^ ; EBOV: anti-EBOV rabbit polyclonal serum^27^; MV-EZ: Abcam [2D7] ab9882; MuV: Abcam [7B10] ab9880; LASV: anti-LASV hyperimmune mouse ascites fluid^28^; ANDV: anti-Pumaala virus nucleoprotein mouse monoclonal antibody^29^; CCHFV: anti-CCHFV hyperimmune mouse ascites fluid^16^.

### Cytopathic effect (CPE) inhibition assays

Inhibition of virus-induced CPE was assayed using CellTiter-Glo 2.0 reagent (Promega) in a HD1 Synergy plate reader. Values were normalized to uninfected cell controls according to % viability as follows: % viability = [(specific value - reference value)/(DMSO control value - reference value)] × 100. Reference values were derived from control wells without cells. Uninfected cell control values (after subtraction of reference values) were set at 100% inhibition of CPE. EC50 values were calculated using four-parameter variable slope non-linear regression fitting of values. NiV-M, NiV-B, HeV, and VSV assays were performed in HeLa cells and H358 cells (NiV-B). AHFV, KFDV, TBEV, and OHFV assays were performed in A549 cells. Cells were seeded at 10,000–20,000 cells per well in white opaque 96-well plates, and compounds were added to the assay plates. Assay plates were transferred to the BSL-4 suite and infected with 0.25-0.5 TCID_50_ per cell, and were assayed with CellTiter-Glo 2.0 between 48–96 hpi.

### Cell viability assay

The CellTiter-Glo 2.0 assay was used to determine viability of uninfected cells treated with 3-fold serial dilutions of the compounds for 72 h. Values were normalized to DMSO controls according to % viability as follows: % viability = [(specific value - reference value)/(DMSO control value - reference value)] × 100. Reference values were derived from control wells without cells. DMSO control values (after subtraction of reference values) were set at 100% viability. 50% viability/cytotoxicity (CC_50_) was calculated using four-parameter variable slope non-linear regression fitting of values.

### NiV minigenome assay

A Nanoluciferase-based NiV minigenome assay was adapted from a previously developed NiV minigenome assay^30^. Briefly, a bacteriophage T7 polymerase-based NiV minigenome was synthesized (Genscript) expressing a reporter fusion construct of Nanoluciferase (Promega) and mNeonGreen fluorescent protein^31^. The open reading frame encoding the reporter fusion protein was flanked by a T7 promoter, hammerhead ribozyme, and NiV leader with N gene untranslated region at the 3′ end, and the L gene untranslated region, NiV trailer, and Hepatitis delta ribozyme at the 5′ end. HeLa cells (5,000 per well) were seeded in 96-well plates, and transfected with appropriate amounts of NiV support plasmids (N (50 ng/well), P (32 ng) and L (50 ng)), HeV minigenome (120 ng) and T7 polymerase (80 ng) prepared in RNase-free TE buffer, mixed with 0.8 μL/well LT-1 transfection reagent (Mirus Bio, Madison, WI) and 10 μL Opti-MEM/well. Complexes were incubated for 30 min at room temperature before adding to cells. Compounds were added directly to the cells 4 h post-transfection. For negative controls, the L plasmid was substituted with an equivalent amount of pcDNA 3.1 plasmid expressing the red fluorescent protein mCherry (Clontech). 48 h post-transfection of minigenome plasmids, 50 μL of Nanoluciferase assay buffer solution (Promega) was added directly to each well. Well contents were transferred to opaque white plates, and after three minutes, luminescence was read on a plate reader (HT-Synergy, Biotek). CellTiter-Glo 2.0 reagent (Promega) was added immediately following reporter minigenome luminescence reading to measure cell viability according to manufacturer’s guidelines. The average raw luminescence value for untreated non-transfected cells was set as 100% cell viability. Percent (%) cell viability for individual wells were calculated by dividing their respective raw luminescence values by the average raw luminescence value for non-transfected cells. Reporter Nanoluciferase activity for each well was then normalized by dividing the nanoluciferase luminescence value by its respective % cell viability as determined above.

### EBOV minigenome assay

A Gaussia luciferase-based EBOV minigenome assay was used^21^. Huh7 cells (10,000 per well) were seeded and transfected with appropriate amounts of EBOV support plasmids (NP (50 ng/well), VP35 (5 ng/well), VP30 (5 ng/well), L (50 ng/well)), EBOV minigenome (150 ng), and T7 polymerase (80 ng) prepared in RNase-free TE buffer mixed with 0.9 μL/well LT-1 transfection reagent (MirusBio) and 10 μL Opti-MEM/well. Complexes were incubated for 30 min at room temperature before adding to cells. Compounds were added directly to the cells 4 h post-transfection. For negative controls, the L plasmid was substituted with an equivalent inactive L plasmid. 48 h post-transfection, 25 μL of supernatant from each well was transferred to opaque white plates, and was combined with 50 μL of Renilla luciferase assay reagent (Promega) via injector and read for bioluminescence in a Biotek HD1 Synergy plate reader following a 0.2 s delay. CellTiter-Glo 2.0 reagent (Promega) was added to the transfected cells, and transferred to a white opaque 96-well plate to measure cell viability according to manufacturer’s guidelines. The average raw luminescence value for untreated non-transfected cells was set as 100% cell viability. Percent (%) cell viability for individual wells were calculated by dividing their respective raw luminescence values by the average raw luminescence value for non-transfected cells. Reporter Gaussia luciferase activity for each well was then normalized by dividing the Gaussia luciferase luminescence value by its respective % cell viability as determined above.

### Infectious virus yield reduction assays

#### EBOV (Makona variant) assay

10,000 Huh7 cells or primary macrophages were infected with 0.25 TCID_50_ per cell or 5 TCID_50_ per cell, respectively for 1 h. Virus inoculum was then removed, cells were washed once with phosphate buffered saline, and replaced with culture medium containing compound in a 10-point 3-fold dilution series. At 72 hpi, supernatants were harvested, serially diluted (10-fold) and mixed with 10,000 Vero cells per well in 96 well plates. At day 5 post-infection, plates were fixed with 10% formalin supplemented with 0.2% Triton-X detergent, and stained with primary rabbit anti-EBOV serum, and after several washes the corresponding anti-rabbit Dylight 488 conjugated secondary antibody was added. Plates were assayed for focus forming units, and quantitated by TCID_50_ assay using the Reed and Muench method^23^.

#### NiV-M assay

10,000 HeLa cells or 5,000 primary HMVEC-L cells were infected with 0.25 TCID_50_ NiV per cell and 1 TCID_50_ per cell, respectively for 1 h. Virus inoculum was then removed, cells were washed once with phosphate buffered saline, and replaced with culture medium containing compound in a 10-point 3-fold dilution series. At 48 hpi supernatants were harvested, serially diluted, and mixed with 10,000 Vero cells per well in 96-well plates. At day 5 post-infection, plates assayed for CPE, and then viral titers were quantitated by TCID_50_ assay using the Reed and Muench method^23^.

#### NiV-B and HeV assay

10,000 HeLa cells were infected with 0.25 TCID_50_ virus per cell. Virus inoculum was left in the growth medium containing compound in a 7-point 3-fold dilution series. At 24 hpi supernatants were harvested, serially diluted, and mixed with 10,000 Vero cells per well in 96-well plates. At day 5 post-infection, plates assayed for CPE, and then viral titers were quantitated by TCID_50_ assay using the Reed and Muench method^23^.

EC_50_ values for all infectious virus yield reduction assays were calculated using four-parameter variable slope non-linear regression fitting of mean values derived from quadruplicate or triplicate samples.

### Sequence similarity clustering analysis

To estimate virus polymerase similarity, we took a hybrid approach that used both structural homology and primary protein sequence information. Distinct ssRNA virus families do not necessarily share a common ancestor in the traditional phylogenetic sense, thus the primary sequence and structure of RNA-dependent RNA polymerases (RdRPs) can be quite divergent. We therefore examined all available viral RdRP crystal structures and utilized secondary structure to align the nucleotide binding domains in iterative fashion by removing non-homologous regions. This resulted in a compelling structural alignment for the nucleotide binding domains (Supplementary Figure 12), although the overall structures of RdRPs were quite divergent. This allowed us to determine functionally homologous nucleotide binding residues (motifs A and B)^18^,^19^,^20^. We extracted the primary amino acid sequences (10 residues each) and aligned them by homology to viral RdRPs without known crystal structures using the global-local pairwise alignment in the Biostrings package in R. Grouping of these sequences was determined by calculating a distance matrix followed by hierarchical linkage clustering. The distance matrix was estimated by taking the negative values of Smith-Waterman alignments (gap open penalty of −5, extension penalty of −1, and match/mismatch values derived from BLOSUM62). Clustering was performed using hclust in R. Results were comparable to those derived from multiple sequence alignments on the extracted regions using MUSCLE^32^ using default parameters.

Crystal structures examined included the following PDB entries:

4WTA (HCV)

5HMZ (Dengue)

2HCN (West Nile)

5A22 (VSV)

4QPX (Norovirus)

1XR5 (Rhinovirus 14)

1XR6 (Rhinovirus 1B)

1TP7 (Rhinovirus 16)

4K4Y (Coxsackie)

3N6M (EV71)

4K4S (Polio)

1RTD (HIV).

## Additional Information

**How to cite this article**: Lo, M. K. *et al*. GS-5734 and its parent nucleoside analog inhibit Filo-, Pneumo-, and Paramyxoviruses. *Sci. Rep.* **7**, 43395; doi: 10.1038/srep43395 (2017).

**Publisher's note:** Springer Nature remains neutral with regard to jurisdictional claims in published maps and institutional affiliations.

## Supplementary Material

Supplementary Figures and Tables

## Figures and Tables

**Figure 1:**
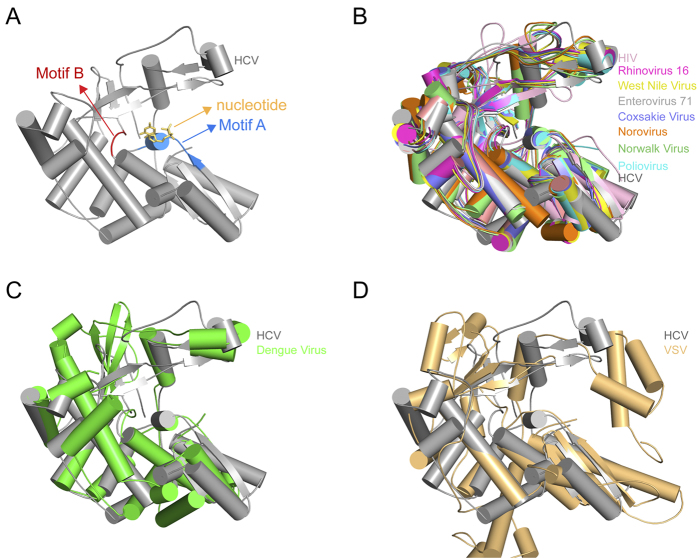
Structural analysis of viral RdRPs nucleotide binding domains. (**A**) Structure of nucleotide binding domain of HCV RdRP showing structural elements (motifs A and B) that interact directly with the nucleotide. (**B**) Structural alignment of nucleotide binding domains of viral RdRPs shows high similarity between numerous structures. (**C**) Structural alignment of HCV and DenV RdRP nucleotide binding domains. (**D**) Structural alignment of HCV and VSV RdRP nucleotide binding domains.

**Figure 2:**
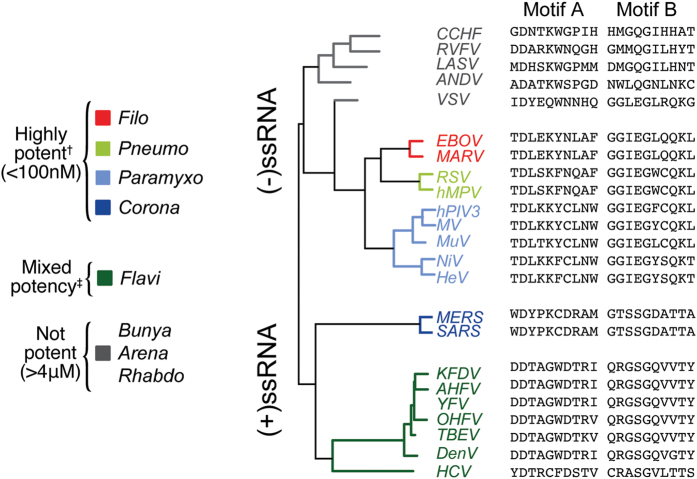
GS-5734 potently inhibits multiple virus families. Clustering by sequence similarity of the nucleotide-interacting regions (motifs A and B) of viral RNA-dependent RNA polymerases correlates with activity of GS-5734 (indicated by color in legend). The tree branches do not necessarily reflect evolutionary relationships (or lack thereof). For details regarding methodology, please refer to methods. ^†^GS-5734 EC_50_ against MuV is higher than 100 nM, with EC_50_ of 0.79 μM. ^‡^GS-5734 is potent (EC_50_ < 4.5 μM) against AHFV, KFDV, TBEV, and OHFV. The parent nucleoside (Nuc) of GS-5734 is also active against DenV and HCV, but not WNV or YFV^3^.

**Table 1:** Mean *in vitro* antiviral activities of Nuc and GS-5734 across 7 virus families.

Virus Family	Virus	Strain	Assay Type	Nuc EC_50_/EC_90_ (μM)/[SI]	GS-5734 EC_50_/EC_90_ (μM)/[SI]
Filo-	EBOV	Rec. Mayinga-GFP	REP	1.6/6.7/[31]	0.066/0.203/[151]
		Rec. Mayinga-Gluc	REP	3.1/11/[16]	0.021/0.053/[476]
		Rec. Makona-ZSG	REP	1.3/3.3/[38]	0.014/0.045/[714]
		Makona	VTR	1.0/2.5/[50]^#^	0.003/0.019/[666]^‡^
	MARV	Rec. Bat371-Gluc	REP	NT	0.019/0.052/[526]
		Rec. Bat371-GFP	REP	1.9/4.6/[26]	0.014/0.047/[714]
Paramyxo-	NiV	Rec. M-Luc2AM	REP	1.5/5.7/[33]	0.045/0.126/[184]
		Rec. M-GFP2AM	REP	2.2/4.0 [22]	0.029/0.053/[286]
		M-1999	VTR	0.49/1.4/[102]^#^	0.047/0.083/[180]^‡^
		B-2004	VTR/CPE	0.83/2.2/[60]^†^	0.032/0.106/[259]
	HeV	1996	VTR/CPE	1.0/1.8/[50]^†^	0.055/0.117/[150]
	hPIV3	Rec. JS-GFP	REP	0.51/1.0/[98]	0.018/0.35/[461]
	MV	Rec. rMV^EZ^GFP(3)	REP	1.0/2.6/[50]	0.037/0.073/[224]
		EZ vaccine	AG	2.0/5.1/[25]	NT
	MuV	IA 2006	AG	9.7/26.3/[5]	0.79/3.4/[10]
Pneumo-	RSV	Rec. rgRSV224 (A2)	REP	0.63/2.2/[79]	0.021/0.059/[395]
	hMPV	Rec. CAN97-83-GFP	REP	0.73/1.7/[NT]	NT
Bunya-	RVFV	Rec. ZH501-GFP	REP	No inhibition	No inhibition
	CCHF	Rec. IbAr 10200	AG	No inhibition	No inhibition
	ANDV	Chile 9717869	AG	NT	7.0/10.1/[1.4]
Arena-	LASV	Josiah	AG	No inhibition	4.5/5.1/[2.2]
Rhabdo-	VSV	New Jersey	CPE	No inhibition	No inhibition
Flavi-	AHFV	200300001	CPE	49.9/ > 150/[NT]	4.2/17.6/[2.4]
	KFDV	P9605	CPE	46.3/ > 350/[NT]	1.8/3.4/[5.6]
	TBEV	Hypr	CPE	51.2/ > 150/[NT]	2.1/3.5/[4.8]
	OHFV	Bogoluvovska	CPE	50.6/ > 350 [NT]	1.2/3.9/[8.3]

VTR: virus titer reduction assay; AG: antigen reduction assay; REP: Reporter assay; CPE: cytopathic effect assay; SI: Selective Index = CC_50_/EC_50_; NT: Not tested; Rec.: recombinant. ^†^>1 log_10_ reduction in virus yield at 24 hours post-infection; ^‡^>4 log_10_ reduction in virus yield at 48 (NiV) or 72 (EBOV) hours post-infection; ^#^>5 log_10_ reduction in virus yield at 48 (NiV) or 72 (EBOV) hours post-infection.
