# Supplementary material for: GS-5734 and its parent nucleoside analog inhibit Filo-, Pneumo-, and Paramyxoviruses
Source: Sci Rep. 2017 Mar 6;7:43395. doi: 10.1038/srep43395 (PMC5338263; doi:10.1038/srep43395)
Supplement: Supplementary Figures and Tables [file srep43395-s1.pdf]

Supplementary Information for:

GS-5734 and its parent nucleoside analog inhibit Filo-, Pneumo-, and Paramyxoviruses

Michael K. Lo[1*], Robert Jordan[2], Punya Shrivastava-Ranjan[1], Anne L. Hotard[1], Mike Flint[1], Laura K. McMullan[1], Dustin Siegel[2], Michael Clarke[2], Richard Mackman[2], Hon Hui[2], Michel Perron[2], Tomas Cihlar[2], Stuart T. Nichol[1], Christina F. Spiropoulou[1*]

[1]Centers for Disease Control and Prevention, Atlanta, Georgia, USA.

[2]Gilead Sciences, Inc. Foster City, California, USA.

*Address for correspondence: Centers for Disease Control and Prevention 1600 Clifton Road, Mailstop G-14, Atlanta, GA 30333 USA

Email: mko2@cdc.gov, ccs8@cdc.gov

Disclaimer: The findings and conclusions in this report are those of the authors and do not necessarily represent those of the Centers for Disease Control and Prevention.

Legends for Supplementary Figures and Tables:

Supplementary Figure 1. Nuc inhibits recombinant fluorescent/luminescent reporter expressing Paramyxoviruses and Filoviruses, but not a reporter-expressing Bunyavirus (RVFV-GFP). Molecular structure of adenosine nucleoside analog GS-441524 (Nuc). Representative dose response curves depicting Nuc antiviral activity against the indicated recombinant reporter viruses. X-axis denotes concentration of Nuc, Y-axis denotes % reporter protein activity. Reporter activity levels derived from DMSO treated infected cells were set as 100% reporter activity. Dose response curves were fitted to the mean value of experiments performed in quadruplicate for each concentration in the 10-point 3-fold dilution series using a 4-parameter non-linear logistic regression curve with variable slope.

Supplementary Figure 2. Nuc inhibits viral antigen production of wild-type Paramxyo- and Filoviruses, but not for Bunya-(CCHFV) or Arenavirus (LASV). Cell-based fluorescence or chemiluminescence immunostaining antigen reduction assay. Representative dose response curves depicting Nuc antiviral activity against the indicated wild-type viruses. X-axis denotes concentration of Nuc, Y-axis denotes % specified viral antigen. Fluorescence/luminescence levels derived from DMSO treated infected cells were set as 100% viral antigen, while levels measured from uninfected cells were set to 0% antigen. Dose response curves were fitted to the mean value of experiments performed in either triplicate or quadruplicate for each concentration in the 7 or 10-point 3-fold dilution series respectively, using a 4-parameter non-linear logistic regression curve with variable slope.

Supplementary Figure 3. Nuc inhibits virus-induced cytopathic effect (CPE) by wild-type Paramxyoviruses, but not for a Rhabdo-(VSV) and minimally for tick-borne flaviviruses (AHFV, KFDV, OHFV, TBEV). Inhibition of virus-induced cytopathic effect was measured using CellTiter-Glo 2.0 assay reagent. Representative dose response curves depicting Nuc antiviral activity against the indicated wild-type viruses. X-axis denotes concentration of Nuc, Y-axis denotes % CPE inhibition. Luminescence levels (indicative of cellular ATP levels as a surrogate marker of cell viability) assayed from DMSO treated uninfected cells were set as 100% CPE inhibition, while levels measured from DMSO treated infected cells were set to 0% CPE inhibition. Dose response curves were fitted to the mean value of experiments performed in either triplicate or quadruplicate for each concentration in the 7 or 10-point 3-fold dilution series respectively, using a 4-parameter non-linear logistic regression curve with variable slope.

Supplementary Figure 4. Nuc inhibits infectious virus production of wild-type NiV and EBOV. Infectious virus yield assay. Virus yield dose response graph depicting Nuc antiviral activity against the indicated wild-type viruses. X-axis denotes concentration of Nuc, Y-axis denotes infectious virus yield by $TCID_{50}$. $TCID_{50}$ values for each data point represent the mean of quadruplicate infections for each concentration in the 10-point 3-fold dilution series of Nuc. Dotted line indicates limit of detection.

Supplementary Figure 5. Nuc does not cause significant cell cytotoxicity. Cell viability assay. Cell viability of Nuc treated uninfected cells was measured at 72 h post-treatment using

CellTiter-Glo 2.0 assay reagent. X-axis denotes concentration of Nuc, Y-axis denotes % Cell viability. Luminescence levels (indicative of cellular ATP levels as a surrogate marker of cell viability) assayed from DMSO treated uninfected cells were set as 100% cell viability.

Supplementary Figure 6. GS-5734 inhibits recombinant fluorescent/luminescent reporter Paramyxoviruses and Filoviruses, but not a reporter Bunyavirus (RVFV-GFP). Molecular structure of phosphoramidate-modified monophosphate adenine nucleotide analog GS-5734. Representative dose response curves depicting GS-5734 antiviral activity against the indicated recombinant reporter viruses. X-axis denotes concentration of GS-5734, Y-axis denotes % reporter protein activity. Reporter activity levels derived from DMSO treated infected cells were set as 100% reporter activity. Dose response curves were fitted to the mean value of experiments performed in quadruplicate for each concentration in the 10-point 3-fold dilution series using a 4-parameter non-linear logistic regression curve with variable slope.

Supplementary Figure 7. GS-5734 inhibits viral antigen production of a wild-type Paramxyovirus (MuV), but not Bunya-(CCHFV, ANDV) or Arenaviruses (LASV). Cell-based fluorescence or chemiluminescence immunostaining antigen reduction assay. Representative dose response curves depicting GS-5734 antiviral activity against the indicated wild-type viruses. X-axis denotes concentration of GS-5734, Y-axis denotes % specified viral antigen. Fluorescence/luminescence levels derived from DMSO treated infected cells were set as 100%, while levels measured from uninfected cells were set to 0%. Dose response curves were fitted to the mean value of experiments performed in quadruplicate for each concentration in the 10-point 3-fold dilution series respectively, using a 4-parameter non-linear logistic regression curve with variable slope.

Supplementary Figure 8. GS-5734 inhibits virus-induced cytopathic effect (CPE) by wild-type Paramxyoviruses, but not for a Rhabdo-(VSV) and minimally for tick-borne flaviviruses (AHFV, KFDV, OHFV, TBEV). Inhibition of virus-induced cytopathic effect was measured using CellTiter-Glo 2.0 assay reagent. Representative dose response curves depicting GS-5734 antiviral activity against the indicated wild-type viruses. X-axis denotes concentration of GS-5734, Y-axis denotes % CPE inhibition. Luminescence levels (indicative of cellular ATP levels as a surrogate marker of cell viability) assayed from DMSO treated uninfected cells were set as 100% CPE inhibition, while levels measured from DMSO treated virus infected cells were set to 0% CPE inhibition. Dose response curves were fitted to the mean value of experiments performed in quadruplicate for each concentration in the 10-point 2-fold or 3-fold dilution series, using a 4-parameter non-linear logistic regression curve with variable slope.

Supplementary Figure 9. GS-5734 inhibits NiV and EBOV minigenome transcription and replication. Minigenome assay. GS-5734. Representative dose response curves depicting GS-5734 antiviral activity against reporter NiV and EBOV minigenomes expressing NanoLuciferase and Gaussia Luciferase, respectively. X-axis denotes concentration of GS-5734, Y-axis denotes % reporter protein activity normalized to levels of cell viability. Reporter activity levels derived from DMSO treated minigenome transfected cells were set as 100% reporter activity. Dose response curves were fitted to the mean value of experiments

performed in quadruplicate for each concentration in the 10-point 3-fold dilution series using a 4-parameter non-linear logistic regression curve with variable slope.

Supplementary Figure 10. GS-5734 inhibits infectious virus production of wild-type NiV and EBOV in primary human lung microvascular endothelial cells and primary human macrophages, respectively. Infectious virus yield assay. Virus yield dose response graph depicting GS-5734 antiviral activity against the indicated wild-type viruses. X-axis denotes concentration of GS-5734, Y-axis denotes infectious virus yield by $TCID_{50}$. $TCID_{50}$ values for each data point represent the mean of quadruplicate infections for each concentration in the 10-point 3-fold dilution series of GS-5734. Dotted line indicates limit of detection of the assay. Mφ- Macrophages; HMVEC-L- Human lung microvascular endothelial cells.

Supplementary Figure 11. GS-5734 induces cell cytotoxicity only at micromolar concentrations. Cell viability assay. Cell viability of GS-5734 treated uninfected cells was measured at 72 h post-treatment using CellTiter-Glo 2.0 assay reagent. X-axis denotes concentration of GS-5734, Y-axis denotes % Cell viability. Luminescence levels (indicative of cellular ATP levels as a surrogate marker of cell viability) assayed from DMSO treated uninfected cells were set as 100% cell viability. Mφ- Macrophages; HMVEC-L- Human lung microvascular endothelial cells.

Supplementary Table 1: Nuc (GS-441524) antiviral activity against multiple virus families. Mean 50% effective inhibition concentration (EC_{50}) values derived from specific assays across indicated number of independent replicate experiments (in parentheses), with standard deviations displayed where applicable.

Supplementary Table 2: GS-5734 antiviral activity against multiple virus families. Mean 50% effective inhibition concentration (EC_{50}) values derived from specific assays across indicated number of independent replicate experiments (in parentheses), with standard deviations displayed where applicable.

Supplementary Table 3: Cell cytotoxicity of Nuc and GS-5734 in various cell types. Mean 50% cytotoxic concentration (CC_{50}) values derived from indicated un-infected cell types treated with either compound for 72 h before assayed for cellular adenosine triphosphate (ATP) levels as an indicator of cell viability using CellTiter-Glo 2.0 assay.

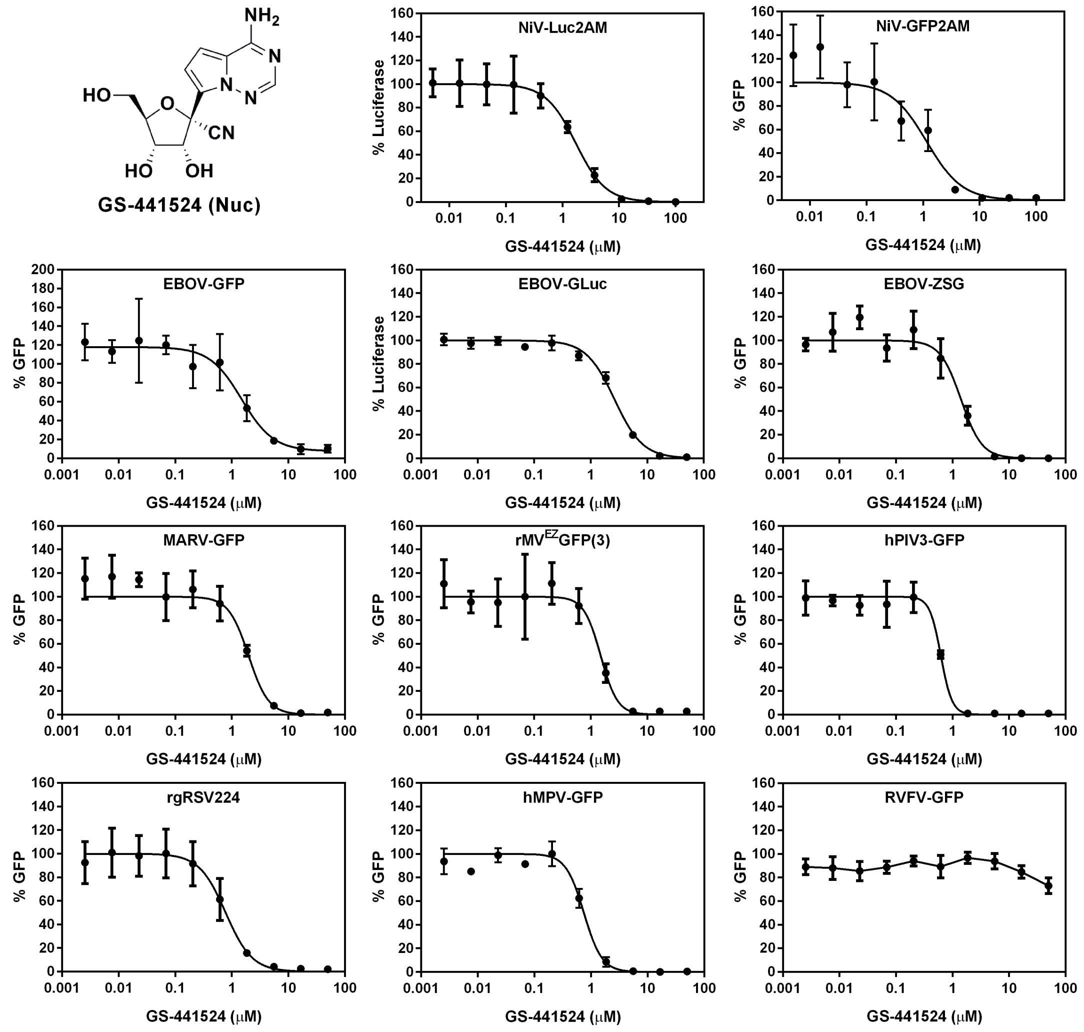
NH2
N
N
N
HO
O
CN
HO
OH
GS-441524 (Nuc)
NiV-Luc2AM
% Luciferase
NiV-GFP2AM
EBOV-GFP
EBOV-GLuc
EBOV-ZSG
MARV-GFP
rMV^EZ GFP(3)
hPIV3-GFP
rgRSV224
hMPV-GFP
RVFV-GFP
% GFP
GS-441524 (μM)

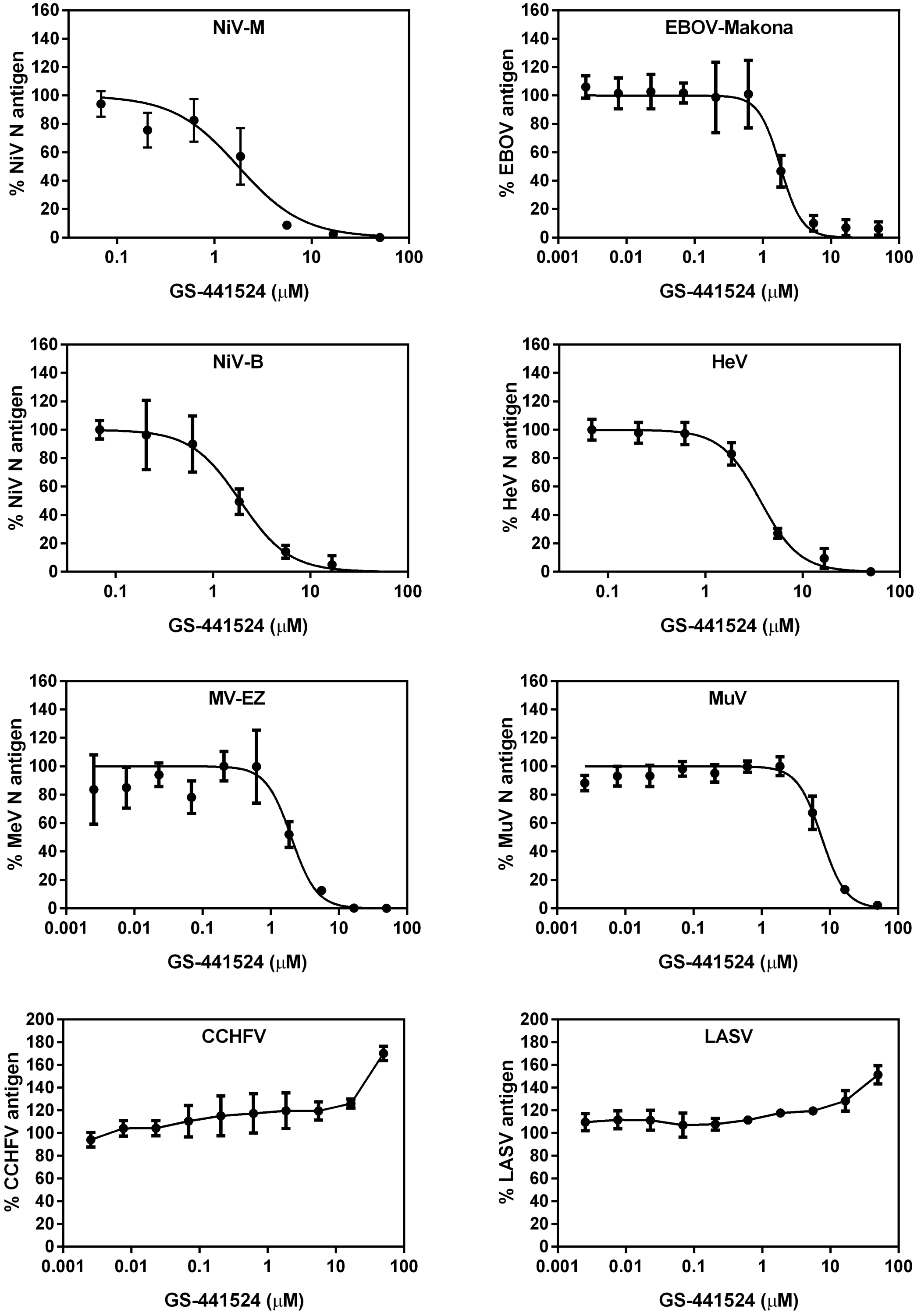
NiV-M
% NiV N antigen
0
20
40
60
80
100
120
140
160
0.1
1
10
100
GS-441524 (μM)
EBOV-Makona
% EBOV antigen
0
20
40
60
80
100
120
140
160
0.001
0.01
0.1
1
10
100
GS-441524 (μM)
NiV-B
% NiV N antigen
0
20
40
60
80
100
120
140
160
0.1
1
10
100
GS-441524 (μM)
HeV
% HeV N antigen
0
20
40
60
80
100
120
140
160
0.1
1
10
100
GS-441524 (μM)
MV-EZ
% MeV N antigen
0
20
40
60
80
100
120
140
160
0.001
0.01
0.1
1
10
100
GS-441524 (μM)
MuV
% MuV N antigen
0
20
40
60
80
100
120
140
160
0.001
0.01
0.1
1
10
100
GS-441524 (μM)
CCHFV
% CCHFV antigen
0
20
40
60
80
100
120
140
160
180
200
0.001
0.01
0.1
1
10
100
GS-441524 (μM)
LASV
% LASV antigen
0
20
40
60
80
100
120
140
160
180
200
0.001
0.01
0.1
1
10
100
GS-441524 (μM)

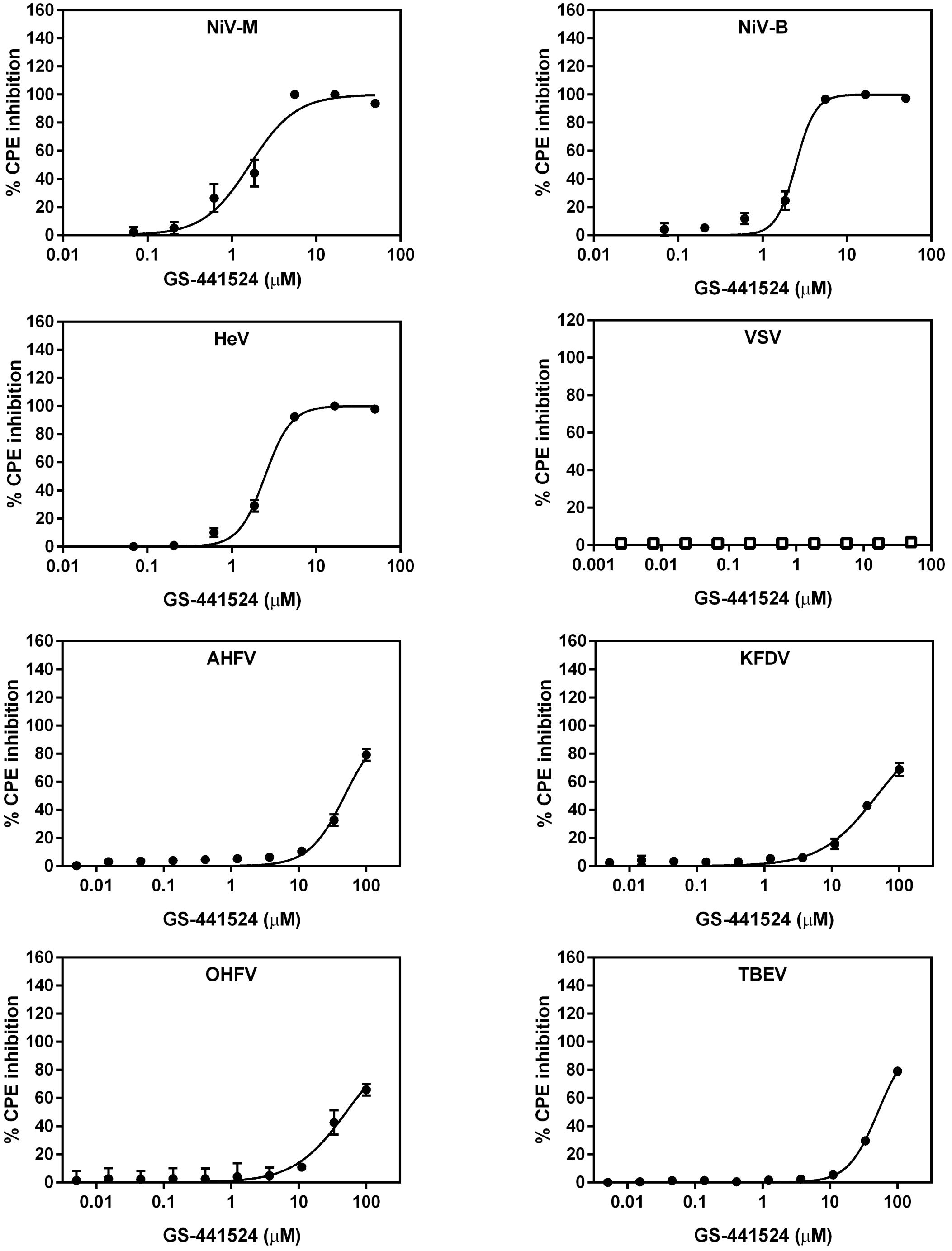

NiV-M
NiV-B
HeV
VSV
AHFV
KFDV
OHFV
TBEV
% CPE inhibition
GS-441524 (μM)

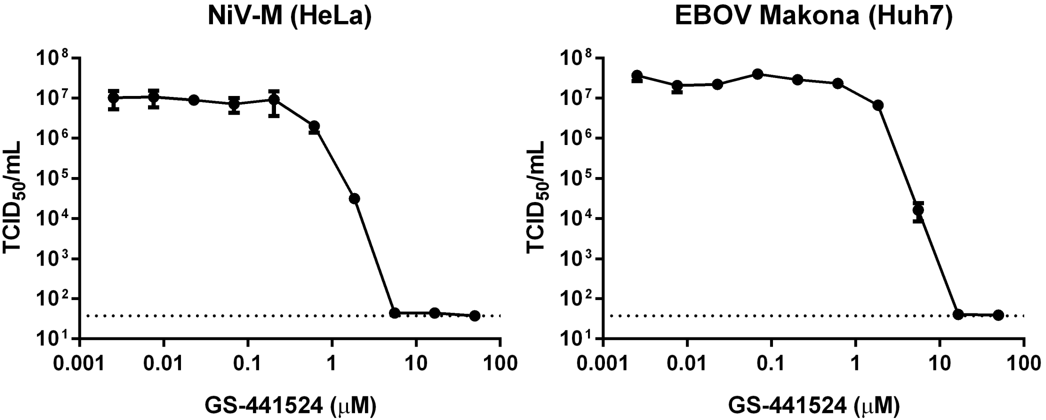
NiV-M (HeLa)
EBOV Makona (Huh7)
TCID50/mL
10^8
10^7
10^6
10^5
10^4
10^3
10^2
10^1
0.001
0.01
0.1
1
10
100
GS-441524 (μM)

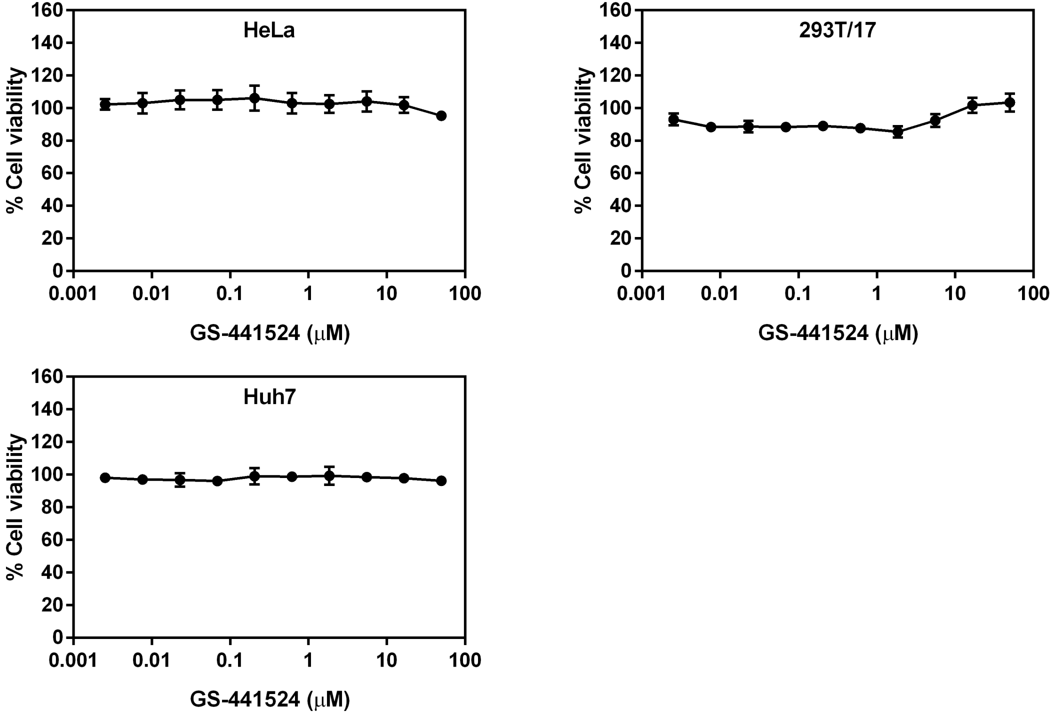
HeLa
293T/17
Huh7
% Cell viability
GS-441524 (μM)
0
20
40
60
80
100
120
140
160
0.001
0.01
0.1
1
10
100

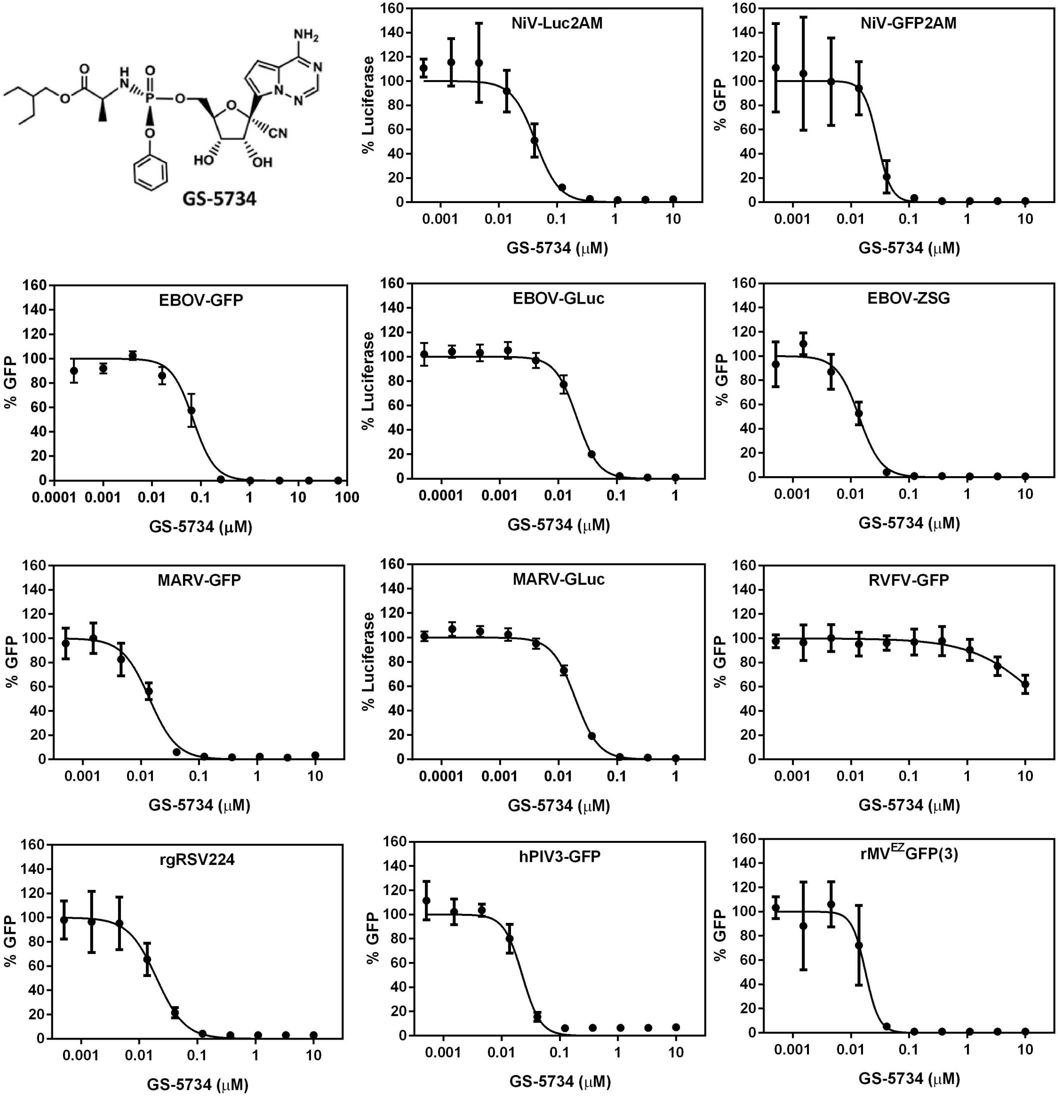

NH2
O
H
N
O
P
O
O
O
N
N
N
CN
HO
OH
O
GS-5734
NiV-Luc2AM
% Luciferase
GS-5734 (μM)
NiV-GFP2AM
% GFP
GS-5734 (μM)
EBOV-GFP
% GFP
GS-5734 (μM)
EBOV-GLuc
% Luciferase
GS-5734 (μM)
EBOV-ZSG
% GFP
GS-5734 (μM)
MARV-GFP
% GFP
GS-5734 (μM)
MARV-GLuc
% Luciferase
GS-5734 (μM)
RVFV-GFP
% GFP
GS-5734 (μM)
rgRSV224
% GFP
GS-5734 (μM)
hPIV3-GFP
% GFP
GS-5734 (μM)
rMV^{EZ}GFP(3)
% GFP
GS-5734 (μM)

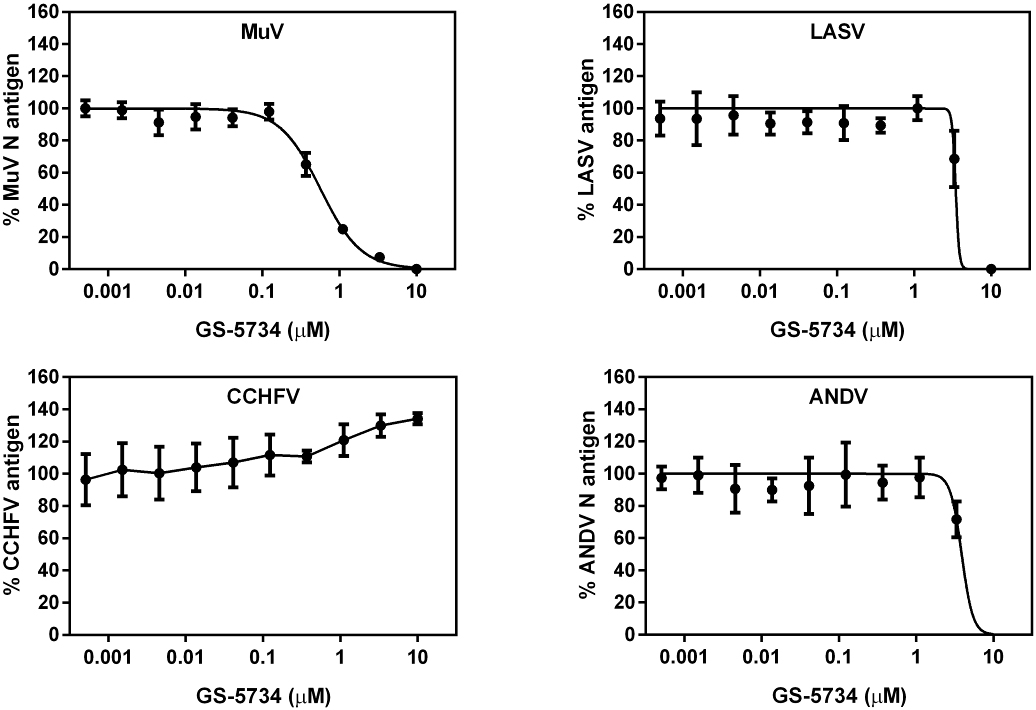
MuV
% MuV N antigen
LASV
% LASV antigen
CCHFV
% CCHFV antigen
ANDV
% ANDV N antigen
GS-5734 (μM)
0.001
0.01
0.1
1
10
0
20
40
60
80
100
120
140
160

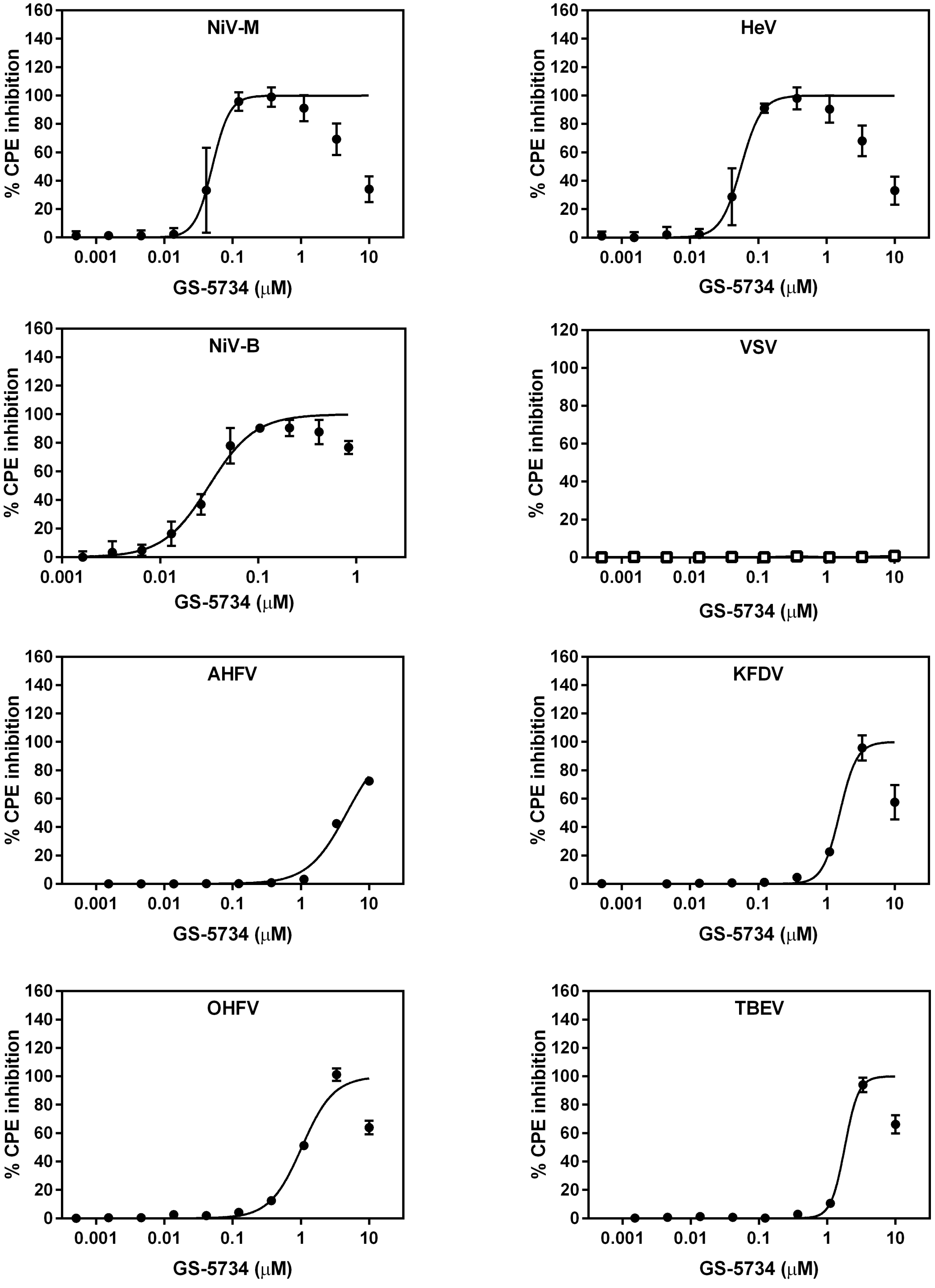
NiV-M
HeV
NiV-B
VSV
AHFV
KFDV
OHFV
TBEV
% CPE inhibition
GS-5734 (μM)
0
20
40
60
80
100
120
140
160
0.001
0.01
0.1
1
10

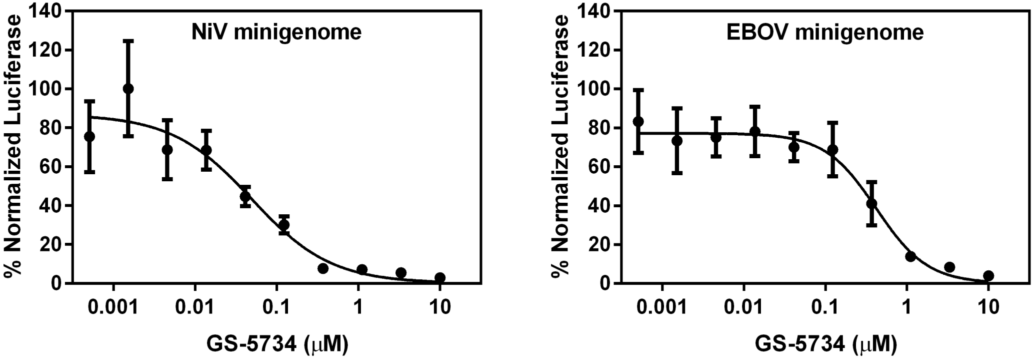
NiV minigenome
EBOV minigenome
% Normalized Luciferase
GS-5734 (μM)
0
20
40
60
80
100
120
140
0.001
0.01
0.1
1
10

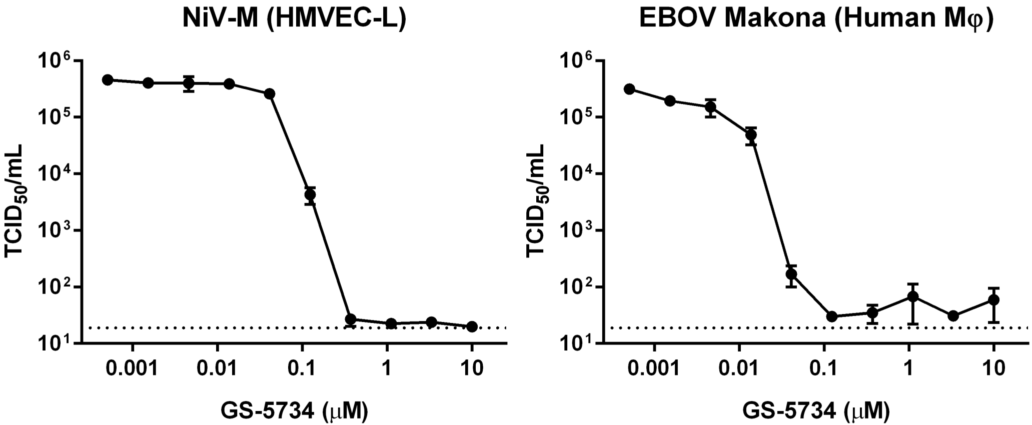

NiV-M (HMVEC-L)
EBOV Makona (Human Mφ)
TCID50/mL
10^6
10^5
10^4
10^3
10^2
10^1
0.001
0.01
0.1
1
10
GS-5734 (μM)

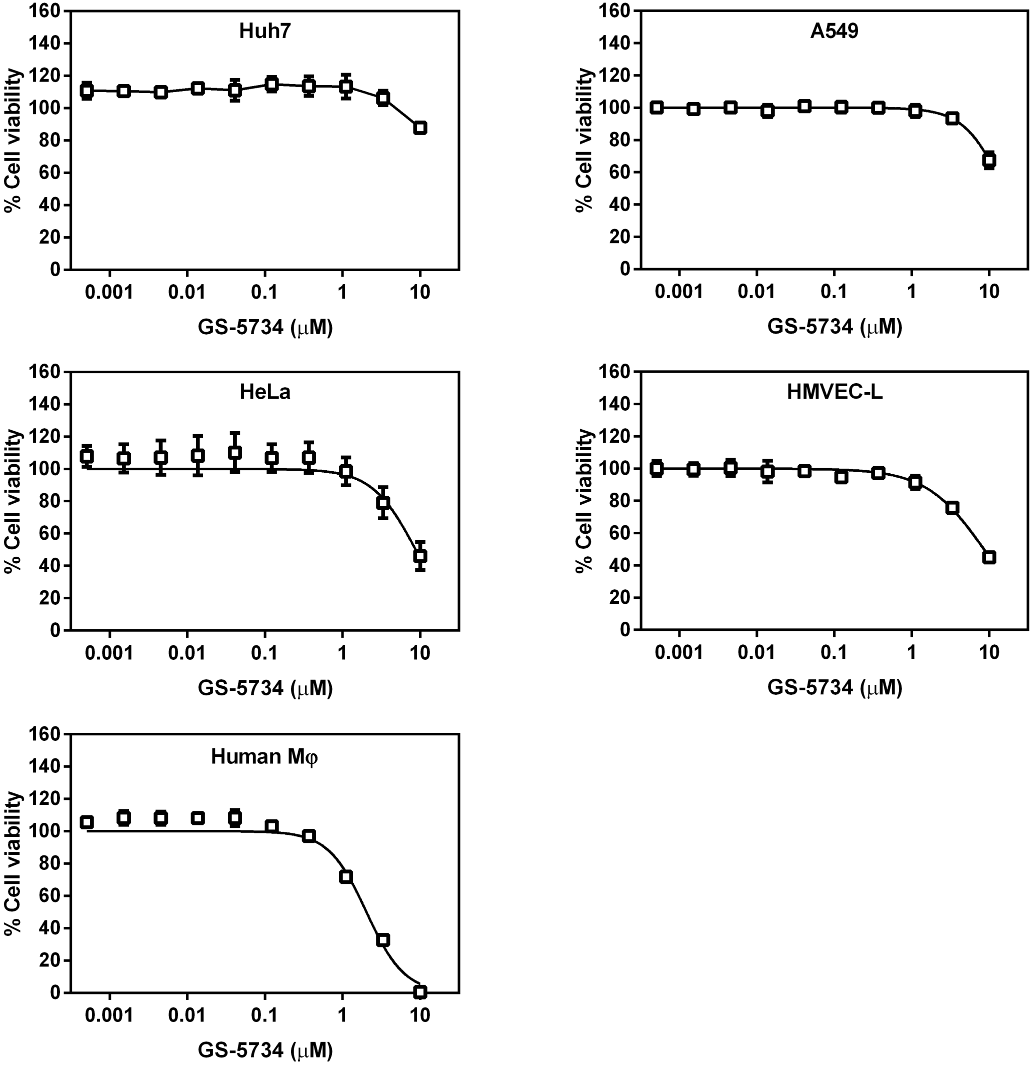
Huh7
A549
HeLa
HMVEC-L
Human Mφ
% Cell viability
GS-5734 (μM)
0
20
40
60
80
100
120
140
160
0.001
0.01
0.1
1
10

			Nuc EC_{50} (μM)			
**Virus Family**	**Virus**	**Strain**	**Reporter assay**	**Antigen Reduction Assay**	**Cytopathic Effect Assay**	**Virus Titer reduction assay**
Filo-	EBOV	Mayinga-GFP	1.61 ± 0.12 (n=2)			
		Mayinga-GLuc	3.1 (n=1)			
		Makona-ZSG	1.28 ± 0.14 (n=2)			
		wt Makona		1.61 ± 0.42 (n=5)		1.02 (n=1)
	MARV	Bat371-GFP	1.86 ± 0.13 (n=2)			
Paramyxo-	NiV	M-Luc2AM	1.57 ± 0.78 (n=9)			
		M-GFP2AM	2.23 ± 1.40 (n=2)			
		M-1999		1.84 ± 0.075 (n=2)	2.12 ± 0.56 (n=3)	0.67 ± 0.18 (n=2)
		B-2004		1.90 (n=1)	2.46 (n=1)	0.52 (n=1)
	HeV	1996		3.68 (n=1)	2.48 ± 0.46 (n=3)	1.00 (n=1)
	hPIV3	JS-GFP	0.52 ± 0.08 (n=2)			
	MV	$rMV^{EZ}GFP(3)$	0.99 ± 0.36 (n=2)			
		EZ vaccine		2.01 (n=1)		
	MuV	IA 2006		9.7 ± 2.1 (n =3)		
Pneumo-	RSV	rgRSV224 (A2)	0.63 ± 0.16 (n=2)			
	hMPV	CAN97-83-GFP	0.73 ± 0.05 (n=4)			
Bunya-	RVFV	ZH501-GFP	> 50 (n=1)			
	CCHF	IbAr 10200		> 50 (n=2)		
	ANDV	Chile 9717869				
Arena-	LASV	Josiah		> 50 (n=2)		
Rhabdo-	VSV	New Jersey			>50 (n=2)	
Flavi-	ALKV	200300001			49.92 ± 2.06 (n=2)	
	KFDV	P9605			46.34 (n=1)	
	TBEV	Hypr			51.24 (n=1)	
	OHFV	Bogoluvovska			50.61 (n=1)	

			GS-5734 EC_{50} (μM)				
**Virus Family**	**Virus**	**Strain**	**Reporter assay**	**Antigen Reduction Assay**	**Cytopathic Effect Assay**	**Virus Titer reduction assay**	**Minigenome assay**
Filo-	EBOV	Mayinga-GFP	0.066 ± 0.004 (n=2)				0.42 (n=2)
		Mayinga-GLuc	0.0207 (n=1)				
		Makona-ZSG	0.0136 ± 0.001 (n=3)				
		Makona				0.0034 (n=1)	
	MARV	Bat 371-GFP	0.0139 ± 0.0003 (n=2)				
		Bat 371-GLuc	0.0193 (n=1)				
Paramyxo-	NiV	M-Luc2AM	0.0449 ± 0.0018 (n=2)				0.049 (n=2)
		M-GFP2AM	0.0287 (n=1)				
		M-1999			0.0655 ± 0.016 (n=2)	0.047 (n=1)	
		B-2004			0.0324 ± .0027 (n=4)		
	HeV	1996			0.0548 ± 0.0013 (n=2)		
	MV	rMV^{EZ}GFP(3)	0.0365 ± 0.028 (n=3)				
		EZ vaccine					
	MuV	IA 2006		0.790 ± 0.117 (n=3)			
	hPIV3	JS-GFP	0.0177 ± 0.0037 (n=3)				
Pneumo-	RSV	A2-GFP	0.0211 ± 0.0011 (n=3)				
Bunya-	RVFV	ZH501-GFP	> 10 (n=1)				
	CCHF	IbAr 10200		> 10 (n=2)			
	ANDV	Chile 9717869		6.95 ± 3.1 (n=2)			
Arena-	LASV	Josiah		4.49 ± 0.48 (n=2)			
Rhabdo-	VSV	New Jersey			> 10 (n=3)		
Flavi-	ALKV	200300001			4.15 ± 0.48 (n=2)		
	KFDV	P9605			1.78 ± 0.22 (n=2)		
	TBEV	Hypr			2.06 ± 0.26 (n=2)		
	OHFV	Bogoluvovska			1.17 ± 0.14 (n=2)		

	Cell Cytotoxicity CC_{50} (μM)	
**Cell ID**	**Nuc**	**GS-5734**
HeLa	>50	8.3
Huh7	>50	>10
A549	NT	>10
293T/17	>50	NT
HMVEC-L	NT	8.5
Mφ	NT	2

NT- not tested
